# Epigenetic Inactivation of Notch-Hes Pathway in Human B-Cell Acute Lymphoblastic Leukemia

**DOI:** 10.1371/journal.pone.0061807

**Published:** 2013-04-26

**Authors:** Shao-Qing Kuang, Zhihong Fang, Patrick A. Zweidler-McKay, Hui Yang, Yue Wei, Emilio A. Gonzalez-Cervantes, Yanis Boumber, Guillermo Garcia-Manero

**Affiliations:** 1 Department of Leukemia, University of Texas M. D. Anderson Cancer Center, Houston, Texas, United States of America; 2 Division of Pediatrics, University of Texas M. D. Anderson Cancer Center, Houston, Texas, United States of America; 3 Department of Internal Medicine, University of Texas Health Science Center at Houston, Houston, Texas, United States of America; 4 Hematology/Oncology Fellowship Program, Division of Cancer Medicine, University of Texas M. D. Anderson Cancer Center, Houston, Texas, United States of America; The Chinese University of Hong Kong, Hong Kong

## Abstract

The Notch pathway can have both oncogenic and tumor suppressor roles, depending on cell context. For example, Notch signaling promotes T cell differentiation and is leukemogenic in T cells, whereas it inhibits early B cell differentiation and acts as a tumor suppressor in B cell leukemia where it induces growth arrest and apoptosis. The regulatory mechanisms that contribute to these opposing roles are not understood. Aberrant promoter DNA methylation and histone modifications are associated with silencing of tumor suppressor genes and have been implicated in leukemogenesis. Using methylated CpG island amplification (MCA)/DNA promoter microarray, we identified Notch3 and Hes5 as hypermethylated in human B cell acute lymphoblastic leukemia (ALL). We investigated the methylation status of other Notch pathway genes by bisulfite pyrosequencing. Notch3, JAG1, Hes2, Hes4 and Hes5 were frequently hypermethylated in B leukemia cell lines and primary B-ALL, in contrast to T-ALL cell lines and patient samples. Aberrant methylation of Notch3 and Hes5 in B-ALL was associated with gene silencing and was accompanied by decrease of H3K4 trimethylation and H3K9 acetylation and gain of H3K9 trimethylation and H3K27 trimethylation. 5-aza-2′-deoxycytidine treatment restored Hes5 expression and decreased promoter hypermethylation in most leukemia cell lines and primary B-ALL samples. Restoration of Hes5 expression by lentiviral transduction resulted in growth arrest and apoptosis in Hes5 negative B-ALL cells but not in Hes5 expressing T-ALL cells. These data suggest that epigenetic modifications are implicated in silencing of tumor suppressor of Notch/Hes pathway in B-ALL.

## Introduction

The Notch receptor signaling pathway has been implicated in regulating hematopoietic stem cell self-renewal, cell lineage commitment, differentiation, and maturation [1], [2], [3]. Human Notch family consists of four Notch receptors (Notch1, 2, 3 and 4) and five ligands (Jagged1/2, Delta-like ligand 1/3/4). Upon ligand binding, the receptors undergo cleavage and release of the intracellular domain, which translocates to the nucleus and associates with the CSL (also known as RBP-Jk) transcription factor. The Notch/CSL complex activates transcription of target genes containing CSL binding elements, most notably members of the Hairy/Enhancer of Split (HES) family (Hes1–6) of transcriptional repressors [4], [5], [6].

During lymphoid development, B- and T-lymphocytes make series of cell fate decisions [7], [8]. Notch signaling has been shown to regulate T and B cell lineage commitment and direct the maturation of T cells at the expense of B cells [9]. Activation of the Notch signaling through point mutations and translocations of the Notch1 gene has been demonstrated in 50–70% of human T cell leukemia/lymphomas [6], [7], [10], [11]. It has also been suggested that nearly all human T cell acute lymphoblastic leukemia (T-ALL) overexpress Notch3 [12]. Constitutive Notch signaling promotes T cell proliferation, results in neoplastic transformation of T lymphoid progenitors, and leads to T cell malignancy. On the other hand, Notch signaling can function as a tumor suppressor in a variety of tissue types [1], [13]. For example, in human B-cell leukemia/lymphoma, constitutive expression of the active forms of the Notch receptors (ICN1-4) or the Notch downstream target gene Hes1 can induce growth arrest and apoptosis [14]. However, the molecular mechanisms underlying the oncogenic and tumor suppressive activities of Notch are not understood.

Appropriate cell lineage determination and differentiation are governed by epigenetic processes such as DNA methylation, histone modification which affect higher order chromatin structure [15]. Methylation of CpG islands in the promoter region of genes is known to correlate with repression of gene transcription [16]. Histone modifications can also act synergistically or antagonistically to define the transcription status of genes [17], [18]. Aberrant promoter CpG island (CGI) methylation and its associated histone modifications are widely accepted mechanisms in silencing tumor suppressor genes and both have been shown to be major contributors and an early events in leukemia pathogenesis [19]. Here we hypothesized that aberrant epigenetic regulation of the Notch-Hes pathway is involved in the pathogenesis of ALL.

## Materials and Methods

### Cell lines and leukemia patient samples

The following human leukemia cell lines were studied: of T cell origin: MOLT4, Jurkat, Peer, T-ALL1, CEM, J-TAG, SupT1 and Loucy; of B cell origin: B-JAB, RS4;11, ALL1, REH, RPMI8226, Raji and Ramos. T-ALL1 and Peer cell lines were obtained from the German Resource Center for Biological Material (DSMZ, Germany). The other cell lines, including 293T, were obtained from the American Type Culture Collection (ATCC). Cell lines were cultured in RPMI 1640 (Invitrogen, Carlsbad, CA) with 10% fetal calf serum (FCS, Gemini Bio-Products, Woodland, CA). Bone marrow (BM) aspiration specimens from patients with B-cell acute lymphoblastic leukemia (B-ALL) and T-ALL were obtained from established tissue banks at MD Anderson Cancer Center (MDACC) following institutional guidelines. This included approval by the MDACC Institutional Review Board (IRB) of both a tissue banking protocol and appropriate laboratory protocol for the proposed studies. Patients signed informed consent for those studies following MDACC IRB guidelines. All samples were collected using Ficoll-Paque density centrifugation. Normal CD19^+^ B cells were collected from 10 healthy volunteers. Consent was also obtained from volunteers. Normal CD19^+^ B cells were isolated using Human B Cell Isolation Kit (Miltenyi Biotec, Auburn, CA) as described. DNA was extracted using standard phenol-chloroform methods.

### DNA bisulfite treatment, Pyrosequencing and Bisulfite Sequencing

Bisulfite induces deamination of unmethylated cytosines, converting unmethylated CpG sites to UpG without modifying methylated sites, as described [19]. For pyrosequencing, a two-step PCR reaction was performed [19]. Primer sequences and conditions are shown in Table S1. The final biotin labeled PCR product was captured by Streptavidin Sepharose HP (Amersham Biosciences, Uppsala, Sweden). PCR products bound to the bead were purified and made single-stranded using a Pyrosequencing Vacuum Prep Tool (Biotage Inc, Uppsala,, Sweden). The sequencing primer was annealed to the single stranded PCR product and pyrosequencing was done using the PSQ HS 96 Pyrosequencing System (Biotage Inc, Uppsala, Sweden). Quantification of cytosine methylation was performed using the PSQ HS96A 1.2 software package (Biotage Inc, Uppsala, Sweden).

Bisulfite sequencing was performed as described to confirm pyrosequencing results in selected samples [19], [20].

### RNA extraction, cDNA synthesis and Real-time PCR

Total cellular RNA was extracted with Trizol (Invitrogen, Carlsbad, CA) and reverse transcribed with MMLV-RT kit (Invitrogen, Carlsbad, CA) and random hexamers. Whole bone marrow (BM) cDNA, CD34^+^ BM cDNA was purchased from Cell Systems (All Cells, LLC. Emeryville, CA). For real-time PCR analysis of mRNA expression, TaqMan probes were purchased from Applied Biosystems and analyzed using an Applied Biosystems Prism 7900 HT Sequence Detection System (Applied Biosystems, Foster City, CA).

### 5-aza-2′-deoxycytidine and/or suberoylanilide hydroxamic acid treatment

To study the effect of epigenetic modulation, leukemia cell lines were cultured in media supplemented with 2 µmol/L of 5-aza-2′-deoxycytidine (DAC) (Sigma, St, Louis, MO) for daily 4 days, 2 µmol/L of DAC for 4 days and then 1 µmol/L suberoylanilide hydroxamic acid (SAHA) (ICN Biomedicals) for the last 24 hours, or 1 µmol/L SAHA for 24 hours alone as described [19].

### Chromatin immunoprecipitation (ChIP) assays

ChIP assays were performed with EZ ChIP™ kit (Millipore Upstate, Charlottesville, VA) according to the manufacturer protocol with modification. Briefly, cross-linked, sonicated DNA products were incubated with anti H3K9Ac, H3K4me3, H3K9me3, H3K27me3 or H3-Ac antibody (Millipore Upstate, Charlottesville, VA) and protein A agarose beads (Millipore Upstate, Charlottesville, VA). Controls were based on manufacturer recommendation. ChIP-real-time PCR analysis was analyzed using an ABI Prism 7900 (Applied Biosystems, Foster City, CA) according to the manufacturer's protocol.

### Luciferase assay

The Hes5 regulatory sequence from 436 bp upstream to 264 bp downstream of the Hes5 transcription start site (TSS) was cloned into luciferase reporter vector pGL3 (Promega Co., Madison, WI). For *in vitro* methylation, the Hes5 promoter was treated with SssI methylase (New England BioLabs, Beverly, MA). 293T (human renal epithelial cell line) cells were transiently transfected with the methylated and unmethylated Hes5–pGL3 constructs or pGL3-basic vector, together with 0.1 ng pRL-TK control vector, which encodes Renilla luciferase (Promega Co., Madison, WI). Luciferase activity was determined using a Dual Luciferase Assay System (Promega Co., Madison, WI). All experiments were carried out in triplicate.

### Lentivirus constructs and gene transduction

Human Hes5 lentiviral construct was generated by inserting human full length Hes5 cDNA (OriGene Technologies, Rockville, MD) into an lentiviral-CMV LTR-Ubiquitin-IRES-GFP transfer vector (FUGW), as described previously [21], [22].

### Statistical analysis

Statistical analyses were performed using Prism 4 (GraphPad Software, Inc.) or Statistica 6 software (Statistica for Windows version 6.0, StatSoft, Tulsa, OK). The Fisher's exact test and t-tests were used to compare gene methylation frequencies or expression levels in leukemia cell lines or leukemia patients and normal control groups. The Spearman non-parametric test was used to determine correlations. All reported p values were 2-sided and *P*<0.05 was considered statistically significant.

## Results

### Identification of hypermethylated Notch3, JAG1, Hes2, Hes4 and Hes5 genes in leukemia cell lines

**Figure 1:**
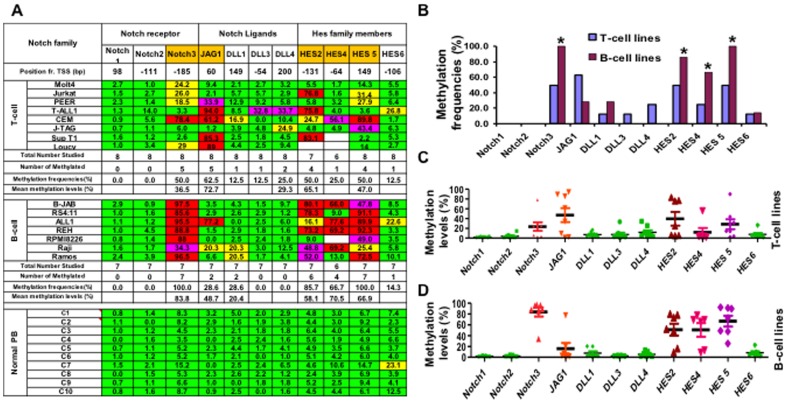
Methylation status of Notch pathway genes in leukemia cell lines and normal peripheral blood cells. **A.** Methylation profile of Notch pathway genes in T cell and B cell leukemia cell lines and normal peripheral blood cells. Bisulfite pyrosequencing was performed to determine the methylation status of Notch pathway genes in 8 T cell and 7 B cell leukemia cell lines and 10 normal peripheral blood cells. Green: methylation density<15%; Yellow: methylation density between 15–29.9%; Pink: methylation density between 30–59.9%, Red box: methylation density >60%. Methylation density >15% was used as the cut off to determine a sample as methylated. Methylation frequency is the percentage of methylated cell lines versus the total number of lines studied for each gene. Position fr. TSS: distance of pyrosequencing sites (bp) away from transcription start site (TSS). **B.** Methylation frequencies of Notch1, Notch2, Notch3, JAG1, DLL1, DLL2, DLL4, Hes2, Hes4, Hes5 and Hes6 genes in leukemia cell lines as detected by pyrosequencing for T cell and B cell lines respectively. *, p<0.05. **C&D.** Methylation densities of Notch1, Notch2, Notch3, JAG1, DLL1, DLL2, DLL4, Hes2, Hes4, Hes5 and Hes6 genes in T and B cell lines respectively. Pyrosequencing data was used to determine methylation density.

Using MCA/DNA promoter microarray, we identified Notch3 and Hes5 as potential methylated targets in primary B-ALL samples [19]. We further investigated the methylation status of Notch pathway genes in a panel of B-ALL and T-ALL cell lines and normal PB cells. Multiple Notch pathway genes were found to contain CpG islands in their promoter regions as identified by using Human Blat program (http://www.genome.ucsc.edu), including receptors (Notch1, Notch2, Notch3), ligands (Jag1, DLL1, DLL3, DLL4) and target genes in the Hes subfamily (Hes2, Hes4, Hes5, Hes6). Their methylation profiles are shown in Figure 1A. Notch3, Hes5, Hes2, Hes4 and JAG1 genes were found frequently hypermethylated in various leukemia cell lines but not in normal controls. Notch3, Hes5, Hes2, Hes4 were methylated more frequently and to a greater extent in B-ALL cell lines while Jag1 was methylated in T-ALL cell lines (Figure 1). Specifically, methylation frequencies of these genes in B-ALL vs. T-ALL were 100 vs. 50% for Notch3 (P<0.05), 86% vs. 50% for Hes5 (P<0.05), 86% vs. 50% for Hes2(P<0.05), 57% vs. 25% for Hes4 ((P<0.05, Figure 1A&B). Methylation density is shown in Figure 1C&D. Significant high density methylation of Notch3 and Hes5 was found in B-ALL cells. The mean methylation density of these two genes in B-ALL vs. T-ALL were 84% vs. 36% for Notch3 and 78% vs. 47% for Hes5. In contrast, Notch1 and Notch2 genes were un-methylated in any of the leukemia cell lines and normal controls, while DLL1, DLL3, DLL4 and Hes6 showed only low levels of methylation (9–27%) in these leukemia cell lines.

### Differential DNA methylation of Notch3 and Hes5 genes in primary B cell leukemia compared to T-ALL

**Figure 2:**
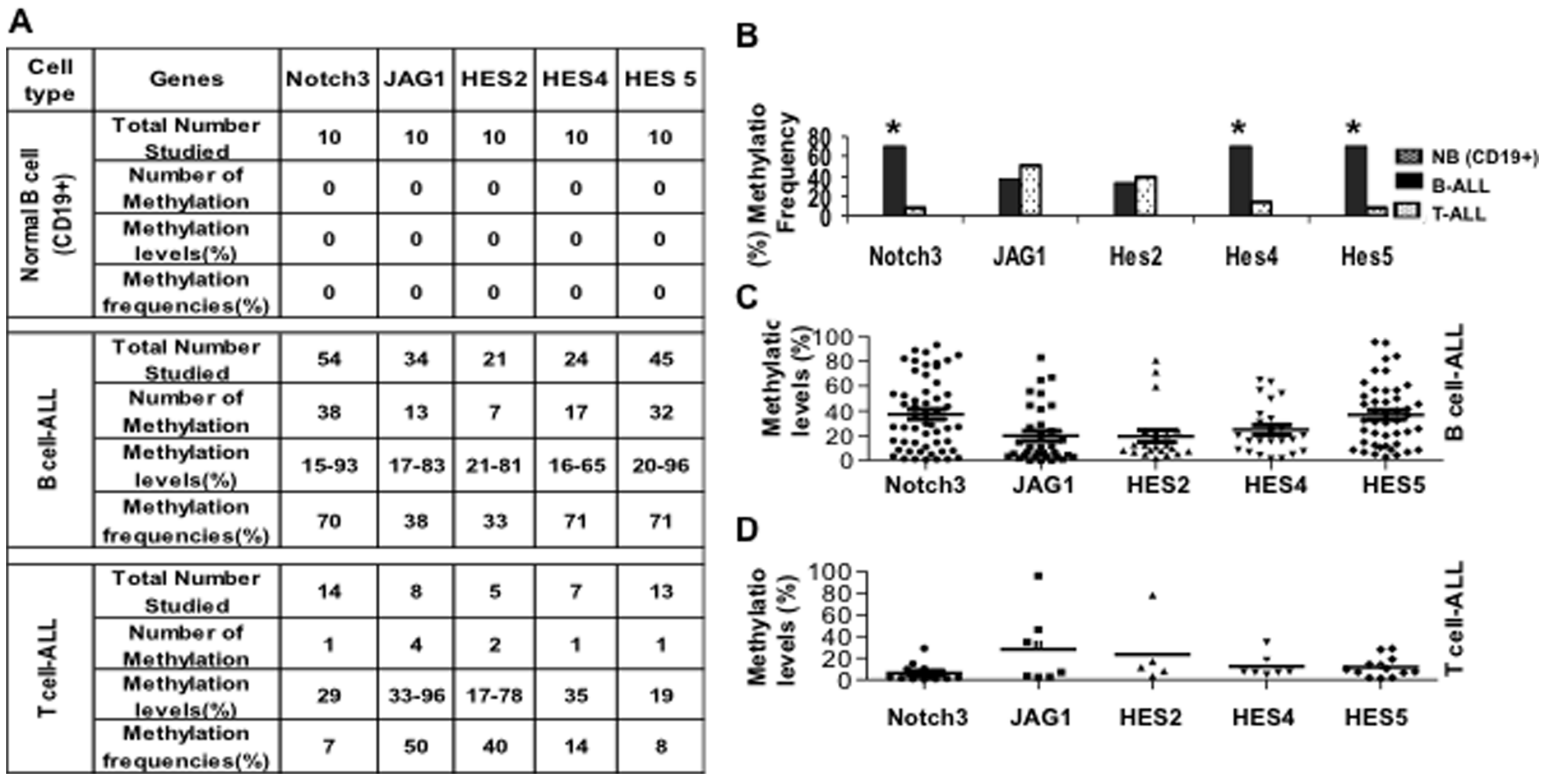
Methylation status of Notch3, JAG1, Hes2, Hes4 and Hes5 genes in normal CD19^+^ B cells, bone marrows from patients with B-ALL and T-ALL. **A & B:** Methylation characteristics of Notch3, JAG1, Hes2, Hes4 and Hes5 genes in normal CD19^+^ B cells, B-ALL and T-ALL as shown by table (A) and figure (B). *, p<0.05. **C&D.** Methylation levels of Notch3, JAG1, Hes2, Hes4 and Hes5 genes in primary B-ALL and T-ALL. Pyrosequencing was performed to determine methylation density. N represents the number of cases in each group.

We subsequently evaluated the methylation status of Notch3, JAG1, Hes2, Hes4 and Hes5 genes in pretreatment patients with different types of ALL. These included 54 patients with B-ALL and 14 with T-ALL. Patient characteristics have been reported previously [19]. To exclude cell lineage specific methylation, we used 10 normal CD19^+^ B cells as controls. Methylation frequencies and densities are shown in Figure 2. As in ALL cell lines, hypermethylation of Notch3 and Hes5 was observed preferentially in primary B-ALL and was much lower in T-ALL (70 vs. 7% and 71 vs. 8% respectively, P<0.05). Hypermethylation of Hes4 occurred more prominently in B-ALL (71 vs. 14%, P<0.05), while Hes2 methylation was similar between groups (33 vs. 40%, P>0.05). Interestingly, hypermethylation of JAG1 was seen to a greater degree in T-ALL than B-ALL patient samples (50 vs. 38%, P<0.05), which is consistent with our findings in ALL cell lines (Figure 1).

### Distinct expression of Notch pathway genes in normal hematopoietic lineage cells

**Figure 3:**
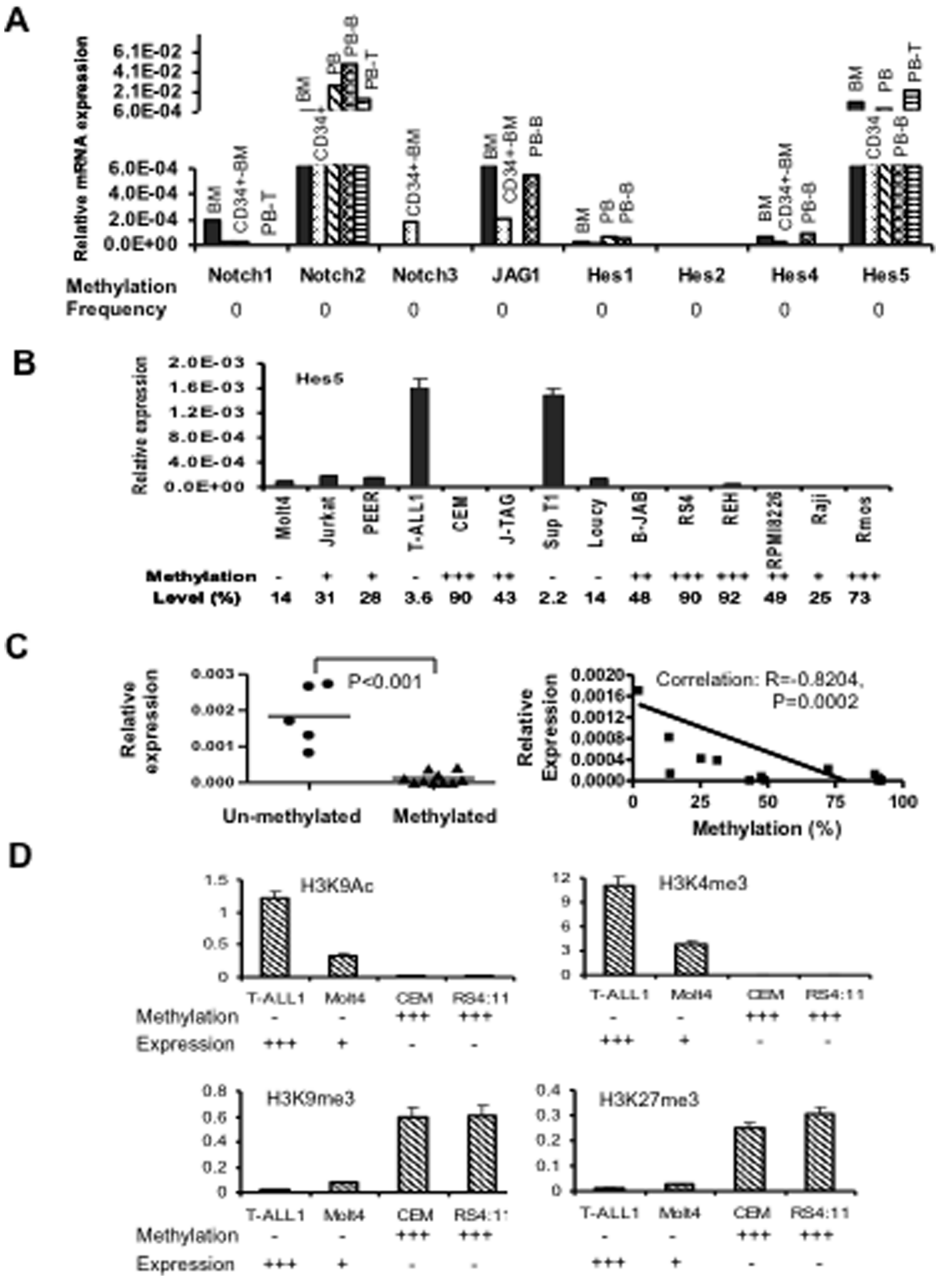
Expression and histone modification analysis of Hes5 genes in leukemia cell lines and normal hematopoietic lineages. **A.** Expression levels of Notch1, Notch2, Notch3, JAG1, Hes1 and Hes5 in normal whole bone marrow (BM), CD34+BM, peripheral blood (PB), CD19^+^ B cells (PB-B) and CD3+ T cells (PB-T). The relative gene expression was determined by real-time PCR assays and normalized to GAPDH. **B.** Hes5 mRNA expression levels in leukemia cell lines by RT-PCR. Hes5 methylation levels from Figure 1A are shown under each cell lines. **C. left.** Hes5 expression is significantly lower in Hes5 methylated cell lines when compared with unmethylated cell lines. **C. right.** Hes5 mRNA expression inversely correlated to methylation level. The solid line represents the regression of the degree of methylation on the Hes5 expression level. **D.** ChIP assay of the Hes5 CpG islands. Chromatin DNA was immunoprecipitated with antibodies against the indicated histone modifications. Immunoprecipitated DNA was amplified by real-time PCR. Percent input was determined as the amount of immunoprecipitated DNA relative to input DNA. Experiments were performed in duplicate. Bars, SD.

To investigate the role of DNA methylation in the regulation of gene expression, mRNA levels of Notch1-3, JAG1, Hes1, Hes2, Hes4 and Hes5 genes were analyzed by quantitative real-time-PCR in normal hematopoietic lineage cells, leukemia cell lines and patients primary bone marrow samples. Figure 3A shows the expression levels of these genes in healthy adult whole bone marrow (BM), CD34^+^ BM cells, whole peripheral blood (PB) cells, PB CD19^+^ B cells (PB-B) cells and PB-T cells. Notch2 and Hes5 transcripts were abundantly detected at all stages of human BM cell development, although the expression level of Hes5 was relatively lower than that of Notch2.

### Dysregulation of Notch pathway gene expression by DNA methylation and histone modification in leukemia cells

We further examined the correlation between the expression levels and methylation status of Notch3, JAG1, Hes2, Hes4 and Hes5 genes in B- and T-ALL cell lines. Hes5, Notch3 and Hes4 genes were either not expressed or very weakly expressed in highly methylated B leukemia cell lines but were abundantly expressed in unmethylated T cell leukemia cell lines such as T-ALL1 and SupT1 (Figure 3B and Figure S1). Hypermethylation of Hes5 CpG islands correlated with down-regulated Hes5 expression as methylation density >15% was used as the cut off to determine a sample as methylated (Figure 3C). We also observed down-regulation of Hes5, Notch3 and Hes4 expression in unmethylated or partially methylated cell lines suggesting that histone deacetylation might be associated with silencing of these genes [18]. To determine the relationship between histone modifications and DNA methylation at the Hes5 locus, we performed ChIP assay in leukemia cell lines having different expression levels of Hes5. We observed that unmethylated and active Hes5 locus in T-ALL1 cells was enriched in H3K9Ac, H3K4me3, but lacked H3K9me3 and H3K27me3 (Figure 3D). In contrast, the hypermethylated and silent Hes5 locus in CEM and RS4;11 cells was hypoacetylated at H3K9Ac and lacked H3K4me3, but was enriched in H3K9me3 and H3K27me3 (Figure 3D). The unmethylated and down-regulated Hes5 locus in Molt4 cells was deacetylated at H3K9Ac and down-regulated H3K4me3, but lacked H3K9me3 and H3K27me3 (Figure 3D). These results indicate that distinct histone modification profiles correlate with Hes5 gene transcriptional activity. Specifically, histone deacetylation and H3K9me is also a feasible mechanism for the silencing of Hes5 in leukemia cells.

### Decitabine treatment restores expression of Notch pathway genes

**Figure 4:**
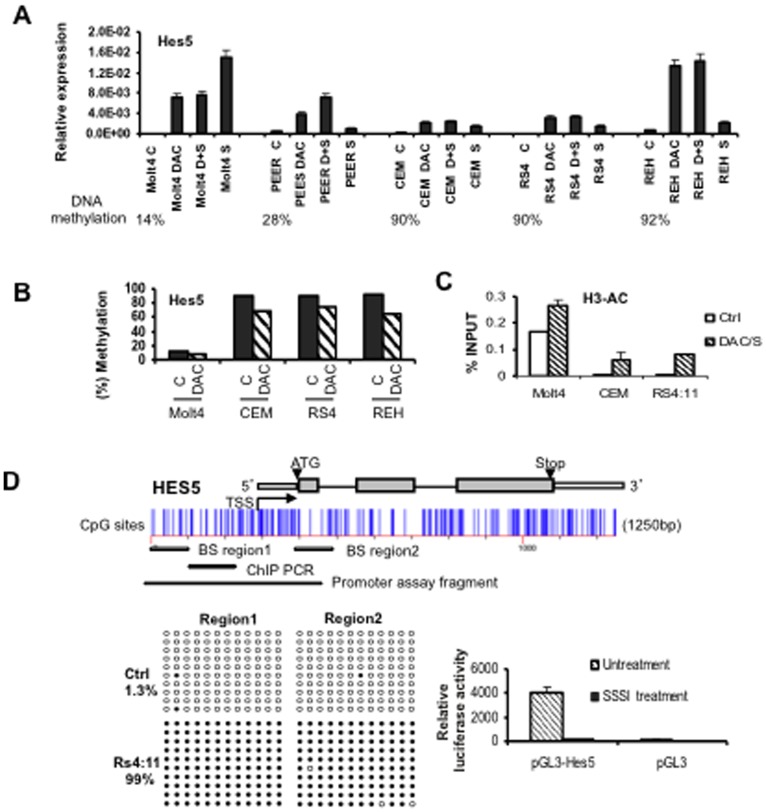
Restoration of Hes5 expression by treatment with 5-aza-2′-deoxycytidine (DAC) and suberoylanilide hydroxamic acid (SAHA) in leukemia cell lines. **A.** Hes5 expression and the effect of demethylating treatment. The leukemia cells were either untreated (C), or treated with DAC only, SAHA only or both (D+S) as described in material and methods. Quantitative RT-PCR was used to measure Hes5 mRNA expression. **B.** Effect of epigenetic modulation on Hes5 gene methylation. Pyrosequencing was performed to determine methylation level. **C.** DAC and SAHA treatment increase acetylated histone H3 as detected by ChIP-real time PCR. **D.** Promoter hypermethylation silences expression of Hes5. **Top.** Diagram of the human Hes5 promoter region studied. CpG sites are indicated by short vertical bars. Arrows point to transcription start site (TSS). The region for bisulfite sequencing, ChIP PCR and promoter activity assay are indicated. **Left bottom:** Methylation analysis of Hes5 gene promoter region by bisulfite sequencing. Each row of circles represents the sequence of an individual clone. Open circles, unmethylated CpG sites; filled circles, methylated CpG sites. **Right bottom:** Promoter activity of the Hes5 CpG islands. The relative luciferase activities of the unmethylated and methylated pGL3-Hes5 constructs in 293T cells.

To explore the role of the DNA methylation in the silencing of gene expression, several cell lines were treated with the demethylating agent decitabine (5-aza-2′-deoxycitidine, DAC) and/or histone deacetylase inhibitor vorinostat (SAHA). In general, expression of Hes5, Hes4 and Notch3 was restored in methylated leukemia cell lines treated by DAC with or without SAHA, a phenomenon associated with gene demethylation (Figure 4A and Figure S2). We also observed an enhancement of Hes5, Hes4 and Notch3 expression in some unmethylated cell lines by SAHA treatment or the combination of DAC and SAHA, suggesting that histone deacetylation is associated with suppressed expression of these genes. We further analyzed Hes5 DNA methylation and histone acetylation status in Molt4, PEER, RS4;11 and REH cell lines before and after DAC treatment. DAC treatment for 5 days or DAC plus SAHA treatment resulted in hypomethylation of Hes5 promoter and hyperacetylation of histone H3 in these cell lines, as measured by bisulfite pyrosequencing and ChIP assay (Figure 4B/C). These data indicates that DNA methylation and histone deacetylation are associated with gene silencing.

### Role of DNA methylation in the transcriptional silencing of Hes5 gene

To test whether the CGI within Hes5 promoter is important for transcription of Hes5, we performed bisulfite sequencing using 159 and 141 bp PCR fragments from −191 to −290, +141 to +203 encompassing 24 CpG sites. Methylation mapping revealed that Hes5 was methylated over the CGI in Hes5 negative RS4;11 cells (Figure 4D). To investigate the role of DNA methylation in regulating Hes5 expression, we tested methylated and unmethylated versions of a Hes5 promoter-driven luciferase reporter and found that the promoter activity of the methylated pHes5-pGL3 construct was 40 times lower (and virtually silenced) than that of the unmethylated construct (Figure 4D). Taken together, these results suggested that promoter hypermethylation of Hes5 gene silences its transcription.

### Distinct expression patterns of Hes5 and Notch3 in primary B cell leukemia compared to T-ALL and their response to 5aza-dC treatment

**Figure 5:**
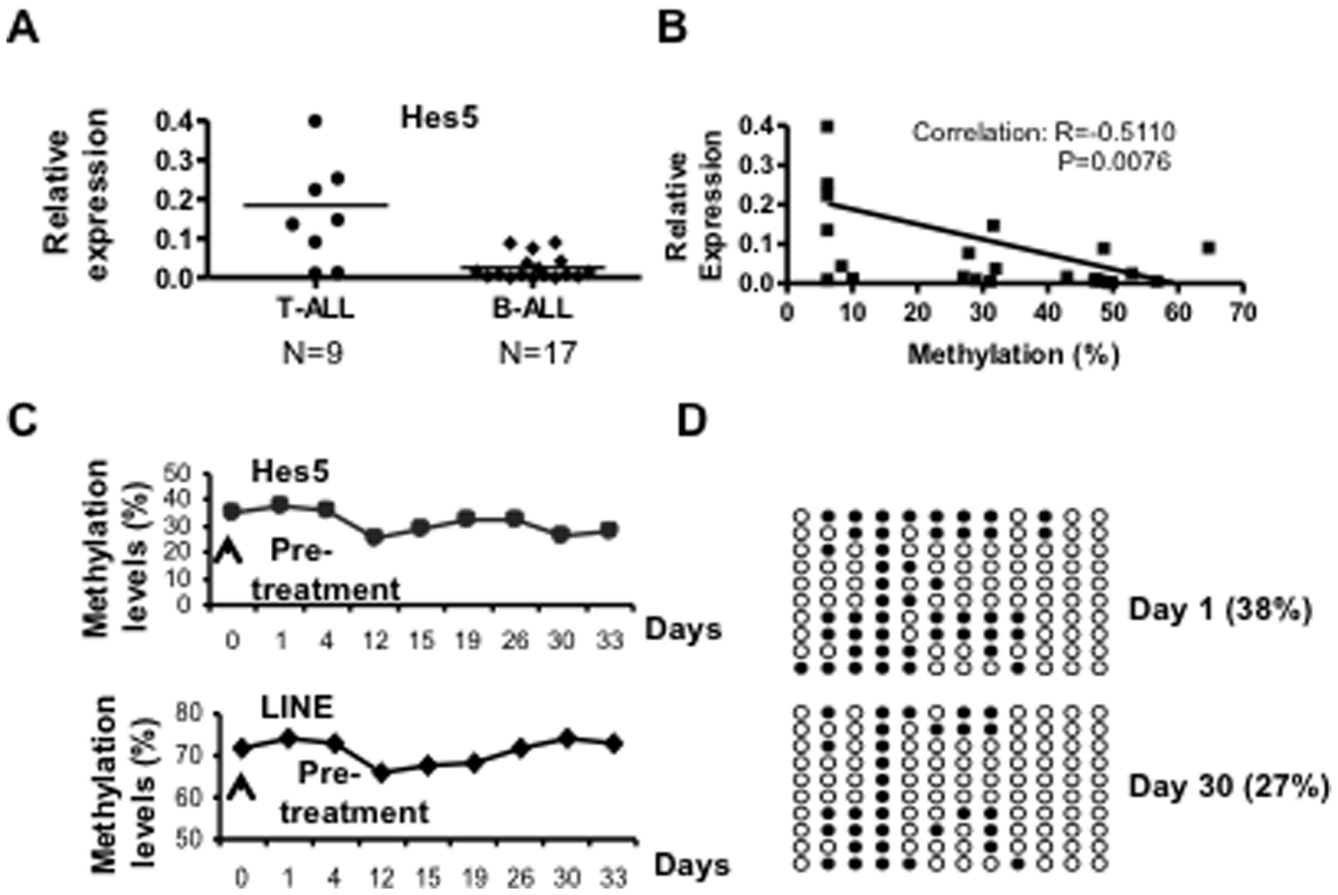
Distinct expression pattern of Hes5 in primary B cell leukemia compared to T-ALL and their response to 5aza-dC (DAC) treatment. **A.** Relative Hes5 mRNA expression in pre-treatment bone marrows from patients with T cell acute lymphoblastic leukemia (T-ALL) and B-ALL, as measured by quantitative RT-PCR, normalized to GAPDH. **B.** Inverse correlation between Hes5 mRNA expression and Hes5 methylation levels in pre-treatment patients, as measured by pyrosequencing. The solid line represents the regression of the degree of methylation on the Hes5 expression level. **C.** Methylation levels of Hes5 and LINE in B-ALL patient at different time points (days 1–35) after DAC treatment, as measured by pyrosequencing. **D.** Bisulfite sequencing map of Hes5 gene from a B-ALL patient at days 1 and 30 of DAC treatment.

To examine Hes5 and Notch3 expression during leukemogenesis, we performed real-time PCR analysis in BM samples from patients with B-ALL and T-ALL. Hes5 and Notch3 were highly expressed in T-ALL, but were significantly decreased or absent in B-ALL samples (Figure 5A and Figure S1). The down-regulation of Hes5 and Notch3 expression correlated with hypermethylation of their CpG islands (Figure 5B and data not shown). We further analyzed Hes5 methylation status in 17 B-ALL patients who received DAC 75 mg/m2 daily for 7 days on an investigational clinical trial (protocol NCT00349596; Garcia-Manero, in preparation). We found substantial reduction in methylation of Hes5 promoter as measured by pyrosequencing in 7 of 14 patients. Methylation analysis for one of them is shown in Figure 5C. For these 7 patients, hypermethylation of Hes5 was confirmed by bisulfite sequencing and the difference on day 30 vs day 1DAC treatment was statistically significant (p<0.05) (Figure 5D). We also analyzed LINE methylation (a surrogate marker of global DNA methylation) dynamics during DAC treatment by pyrosequencing. We found a significant decrease in global methylation by day 12 (Figure 5C bottom), which was similar to the Hes5 methylation pattern (Figure 5C top).

### Hes5 inhibits proliferation and induces apoptosis in B cells but not in T cells

**Figure 6:**
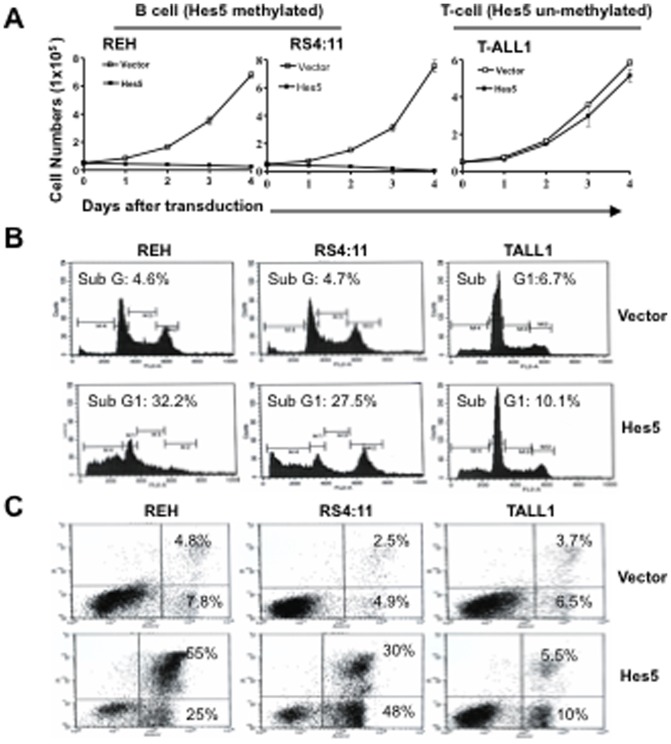
Hes5 inhibits proliferation and induces apoptosis in B cells but not in T cells. **A.** REH, RS4;11 and T-ALL1 cell lines were transduced with lentivirus expressing Hes5 or empty vector. Cell numbers were measured on day using trypan blue exclusion assay. **B.** Cell cycle distributions measured 2 days after lentivirus infection using propidium iodide (PI) staining to measure DNA content. The percentage of cells in sub-G1 (<2N DNA) is presented. **C.** Analysis of apoptosis 3 days after lentivirus infection using flow cytometry, PI staining and annexinV staining.

To assess the effect of Hes5 restoration in leukemia cells, we transduced FUGW-Hes5 lentiviral constructs into two Hes5 methylated/silenced B cell lines REH and RS4;11, and one Hes5 expressing T cell line T-ALL1. A GFP only lentivirus was used as a control. Hes5 transgene expression was confirmed by western blot (Figure S3). Hes5 transgene dramatically suppressed the growth rate of both Hes5 transduced REH and RS4;11 cell lines. Conversely, no significant effects were observed in T-ALL1 cells infected with Hes5 lentivirus. No significant alterations in cells infected with FUGW-GFP vector (Figure 6B). We also performed flow cytometry analysis of these cells 2 days after lentiviral transduction. Both Hes5 infected REH and RS4;11 cells displayed a significant appearance of a sub-G1 fraction. In contrast, no significant changes were observed in both cell lines infected with empty vector. No significant alterations in the cell cycle profile were observed in T-ALL1 cell lines infected with Hes5 or empty vector (Figure 6C). We further performed AnnexinV staining. The Hes5 transduced REH and RS4;11 cells demonstrated significant increase of apoptotic cells (80% and 78% in FUGW-Hes5 REH and RS4;11 cells, respectively) 3 days after transduction, whereas only 13% and 7% of empty vector transduced REH and RS4;11 cells stained positively for AnnexinV (Figure 6C). No significant alterations in the AnnexinV staining were observed in T-ALL1 cell lines infected with Hes5 or empty vector (Figure 6C).

## Discussion

Leukemia is both a genetic and epigenetic disease. Abnormal promoter DNA methylations and histone modifications have gained increasing recognition as an important mechanism for silencing of tumor suppressor genes and contribute to leukemogenesis along with genetic alterations [23]. Using MCA/microarray, we identified Notch pathway genes Notch3 and Hes5 as hypermethylated in human B-ALL samples. In this study, we investigated the methylation status of Notch pathway genes in leukemia cell lines and patient samples by pyrosequencing. Methylation verification revealed that Notch3, Hes5, Hes2, Hes4 and JAG1 genes were frequently hypermethylated in various leukemia cell lines but not in normal controls. Methylation analysis of these genes were different in various types of leukemias. JAG1, Hes2 and Hes4 were commonly methylated in various leukemia cell lines and primary B-ALL and T-ALL but not in normal CD19+ B cells. In contrast, Notch3 and Hes5 were found preferentially hypermethylated in B-lineage lymphoblastic cell lines and primary B-ALL, but methylated at very lower levels or unmethylated in T cell lines or primary T-ALL. In most cases, Notch3, Hes4 and Hes5 are found to be coordinately methylated. The observation of concomitant methylation of several Notch pathway genes at different chromosomal loci suggests that epigenetic disruption of Notch signaling may be an important event in leukemia pathogenesis. The distinct methylation pattern of Notch3 and Hes5 genes in primary B cell leukemia compared to T-ALL further suggest that aberrant DNA methylation occur in a tumor specific and lineage-specific fashion.

In the present study, we also investigated the expression patterns of Notch pathway genes in normal hematopoietic lineage cells. We demonstrated that Notch2 and Hes5 were highly expressed in multiple lineages, whereas Notch3 was not expressed in mature lymphocytes, but was expressed on a subset of CD34+ stem/progenitor cells in BM. These expression patterns imply that the different Notch genes may have very distinct functions during hematopoiesis and that Notch3 could be a specific regulator of stem cell development.

We further examined the expression levels of Notch pathway genes on primary leukemia cell blasts and leukemia cell lines. Notch3 and Hes5 genes were predominantly expressed in primary T-ALL and some T cell lines but were silenced in majority of B cell leukemia and B cell lines, suggesting that Notch3 and Hes5 could be used as T cell lineage specific markers for leukemia diagnosis. We demonstrated a leukemia specific hypermethylation and aberrant histone modifications in transcriptional silencing Notch pathway gene expression. All normal CD19^+^ B cells were completely unmethylated at the Notch3, Hes2, Hes4 and Hes5 CpG islands, excluding the possibility that cell lineage specific methylation accounted for the observed mehylation in B-ALL. Most importantly, hypermethylation and histone deacetylation of Notch pathway gene correlated with down-regulation of gene expression. The transcriptionally active Hes5 locus in T-ALL1 cells was unmethylated, hyperacetylated at H3K9 and hypermethylated at H3K4. In contrast, the silent Hes5 locus in CEM and RS4;11 cells was hypermethylated, hypoacetylated at H3K9Ac and hypomethylated at H3K4, but was hypermethylated at H3K9 and H3K27. We established a further link between Notch pathway gene CpG islands hypermethylation and their gene silencing by demethylation treatment. DNA demethylating agent DAC and histone deacetylation inhibitor SAHA treatment restored the expression of the Notch pathway genes in several hypermethylated and silenced cell lines. In addition, the CpG sites around the Hes5 promoter region, whose methylation was associated with the silencing of this gene in B cell lines, showed clear promoter activity. Therefore, DNA hypermethylation, as well as histone deacetylation and methylation are potential mechanisms of inactivation of Notch pathway genes in leukemias. However, some cell lines showed reduced Hes5 expression without DNA methylation, and the effect of DAC alone or with SAHA increased Hes5 expression, suggesting that histone modification rather than DNA methylation contributed to the silencing of Hes5.

To further confirm the importance of epigenetic mechanisms in down-modulation of negative growth regulatory genes and tumorigenesis [24], we re-expressed human Hes5 in leukemia cell lines with or without Hes5 methylation. Forced restoration of Hes5 resulted in cell growth inhibition and apoptosis only in Hes5 methylated and silenced B-ALL lines (REH and RS4;11) but not in Hes5 unmethylated and expressing T cell lines. These findings are of functional significance as epigenetic suppression of Notch pathway genes may be critical to disrupt their role in Notch signaling, allowing uncontrolled proliferation and apoptosis-resistance contributing to leukemia progression. It is also consistent with the model that activated Notch may function as either an oncogenic factor in T cell leukemia or a tumor suppressor in B cell leukemia/lymphoma [1], [13]. It appears that the dual and opposing function of Notch signaling is cell lineage and cell context specific, and is possibly controlled by epigenetic regulation of Notch pathway gene expression in different cell types. Because several Notch pathway genes exhibit tumor suppressor function in B-ALL cells [14], the epigenetic silencing of the Notch signaling pathway may provide a selective growth advantage to leukemia cells. That said, one of the limitations of this study is that we have not elucidated the mechanisms for differential induction of apoptosis.

In summary, this is the first report that multiple members of the Notch pathway are commonly hypermethylated and down-regulated in human leukemia cell lines and primary B cell leukemias. We show distinct methylation and expression patterns of Notch3 and Hes5 in B cell leukemias compared with T-ALL. Treatment of leukemia cells with the demethylation and deacetylation agents induced expression of these pathway genes. Our study suggest that epigenetic regulation of Notch pathway gene expression correlated with their distinct function in human B versus T cell leukemias and strengthen the observation that some Notch pathway genes may function as tumor suppressors in B cell leukemias, being down-regulated by DNA methylation. This tumor suppressive properties are consistent with recently reported role of Notch pathway in myeloid leukemia [25] and confirm prior results on the tumor suppressive nature of Notch signaling, including Notch3, in B cell malignancies [14]. Our findings of coordinate down-regulation of multiple members of the Notch pathway through epigenetic remodeling may have major implications for the future understanding of leukemia initiation and progression. Further evaluation of epigenetic effects on the Notch pathway and other pathways of growth regulation may provide novel therapeutic approaches for the treatment of leukemias.

## Supporting Information

Figure S1 **Expression of Notch3, JAG1, Hes4 and Hes2 in normal bone marrow (BM), CD34+ BM, BMs from patients with T cell acute lymphoblastic leukemia (T-ALL), B-ALL, and various leukemia cell lines.** The relative gene expression was determined by real-time PCR assays and normalized to that of GAPDH.
(PPT)

Figure S2 **Notch3, Hes4, Hes2 and Hes6 expression levels in various leukemia cell lines.** The leukemia cells were either untreated, or treated with 5-aza-2′-deoxycytidine (DAC) only, suberoylanilide hydroxamic acid (SAHA) only or both (D+S) as described in material and methods. Real-time PCR analysis. In general, expression of Notch3 and Hes4 was restored in some leukemia cell lines treated by DAC with or without SAHA. Hes2 was un-respond to any DAC, and SAHA treatment. In contrast, Hes6 was respond to DAC, and SAHA treatment.
(PPT)

Figure S3 **A. FUGW lentiviral constructs for transducing Hes5 and controls.** B. Western blot analysis. Hes5 expression was detected in untreated T-ALL1 cells, as well as 293T and TALL1 cells transduced with Hes5.
(PPT)

Table S1 **Primer sequences used for bisulfite pyrosequencing, ChIP assay and Hes5 promoter cloning.**
(PPT)

## References

[1] Dotto GP (2008) Notch tumor suppressor function. Oncogene 27: 5115–5123. 18758480 10.1038/onc.2008.225 PMC2747622

[2] Jundt F, Schwarzer R, Dorken B (2008) Notch signaling in leukemias and lymphomas. Curr Mol Med 8: 51–59. 18289013 10.2174/156652408783565540

[3] Singh N, Phillips RA, Iscove NN, Egan SE (2000) Expression of notch receptors, notch ligands, and fringe genes in hematopoiesis. Exp Hematol 28: 527–534. 10812242 10.1016/s0301-472x(00)00146-6

[4] Choi JH, Park JT, Davidson B, Morin PJ, Shih Ie M, et al (2008) Jagged-1 and Notch3 juxtacrine loop regulates ovarian tumor growth and adhesion. Cancer Res 68: 5716–5723. 18632624 10.1158/0008-5472.CAN-08-0001 PMC2562671

[5] Katoh M, Katoh M (2007) Integrative genomic analyses on HES/HEY family: Notch-independent HES1, HES3 transcription in undifferentiated ES cells, and Notch-dependent HES1, HES5, HEY1, HEY2, HEYL transcription in fetal tissues, adult tissues, or cancer. Int J Oncol 31: 461–466. 17611704

[6] Weng AP, Ferrando AA, Lee W, Morris JPt, Silverman LB, et al (2004) Activating mutations of NOTCH1 in human T cell acute lymphoblastic leukemia. Science 306: 269–271. 15472075 10.1126/science.1102160

[7] Ellisen LW, Bird J, West DC, Soreng AL, Reynolds TC, et al (1991) TAN-1, the human homolog of the Drosophila notch gene, is broken by chromosomal translocations in T lymphoblastic neoplasms. Cell 66: 649–661. 1831692 10.1016/0092-8674(91)90111-b

[8] Walter K, Bonifer C, Tagoh H (2008) Stem cell-specific epigenetic priming and B cell-specific transcriptional activation at the mouse Cd19 locus. Blood 112: 1673–1682. 18552207 10.1182/blood-2008-02-142786

[9] He Y, Pear WS (2003) Notch signalling in B cells. Semin Cell Dev Biol 14: 135–142. 12651097 10.1016/s1084-9521(02)00182-9

[10] Leong KG, Karsan A (2006) Recent insights into the role of Notch signaling in tumorigenesis. Blood 107: 2223–2233. 16291593 10.1182/blood-2005-08-3329

[11] Aster JC, Pear WS, Blacklow SC (2008) Notch signaling in leukemia. Annu Rev Pathol 3: 587–613. 18039126 10.1146/annurev.pathmechdis.3.121806.154300 PMC5934586

[12] Talora C, Cialfi S, Oliviero C, Palermo R, Pascucci M, et al (2006) Cross talk among Notch3, pre-TCR, and Tal1 in T-cell development and leukemogenesis. Blood 107: 3313–3320. 16368887 10.1182/blood-2005-07-2823

[13] Zweidler-McKay PA (2008) Notch signaling in pediatric malignancies. Curr Oncol Rep 10: 459–468. 18928660 10.1007/s11912-008-0071-2

[14] Zweidler-McKay PA, He Y, Xu L, Rodriguez CG, Karnell FG, et al (2005) Notch signaling is a potent inducer of growth arrest and apoptosis in a wide range of B-cell malignancies. Blood 106: 3898–3906. 16118316 10.1182/blood-2005-01-0355 PMC1895093

[15] Ivascu C, Wasserkort R, Lesche R, Dong J, Stein H, et al (2007) DNA methylation profiling of transcription factor genes in normal lymphocyte development and lymphomas. Int J Biochem Cell Biol 39: 1523–1538. 17433759 10.1016/j.biocel.2007.02.006

[16] Garcia-Manero G, Yang H, Kuang SQ, O'Brien S, Thomas D, et al (2009) Epigenetics of acute lymphocytic leukemia. Semin Hematol 46: 24–32. 19100365 10.1053/j.seminhematol.2008.09.008 PMC3833728

[17] Kondo Y, Shen L, Cheng AS, Ahmed S, Boumber Y, et al (2008) Gene silencing in cancer by histone H3 lysine 27 trimethylation independent of promoter DNA methylation. Nat Genet 40: 741–750. 18488029 10.1038/ng.159

[18] Kapoor-Vazirani P, Kagey JD, Powell DR, Vertino PM (2008) Role of hMOF-dependent histone H4 lysine 16 acetylation in the maintenance of TMS1/ASC gene activity. Cancer Res 68: 6810–6821. 18701507 10.1158/0008-5472.CAN-08-0141 PMC2585755

[19] Kuang SQ, Tong WG, Yang H, Lin W, Lee MK, et al (2008) Genome-wide identification of aberrantly methylated promoter associated CpG islands in acute lymphocytic leukemia. Leukemia 22: 1529–1538. 18528427 10.1038/leu.2008.130

[20] Shu J, Jelinek J, Chang H, Shen L, Qin T, et al (2006) Silencing of bidirectional promoters by DNA methylation in tumorigenesis. Cancer Res 66: 5077–5084. 16707430 10.1158/0008-5472.CAN-05-2629

[21] Wang Z, Miura N, Bonelli A, Mole P, Carlesso N, et al (2002) Receptor tyrosine kinase, EphB4 (HTK), accelerates differentiation of select human hematopoietic cells. Blood 99: 2740–2747. 11929761 10.1182/blood.v99.8.2740

[22] Kuang SQ, Ling X, Sanchez-Gonzalez B, Yang H, Andreeff M, et al (2007) Differential tumor suppressor properties and transforming growth factor-beta responsiveness of p57KIP2 in leukemia cells with aberrant p57KIP2 promoter DNA methylation. Oncogene 26: 1439–1448. 16936778 10.1038/sj.onc.1209907

[23] Roman-Gomez J, Jimenez-Velasco A, Barrios M, Prosper F, Heiniger A, et al (2007) Poor prognosis in acute lymphoblastic leukemia may relate to promoter hypermethylation of cancer-related genes. Leuk Lymphoma 48: 1269–1282. 17613754 10.1080/10428190701344899

[24] Yendamuri S, Trapasso F, Ferracin M, Cesari R, Sevignani C, et al (2007) Tumor suppressor functions of ARLTS1 in lung cancers. Cancer Res 67: 7738–7745. 17699778 10.1158/0008-5472.CAN-07-1481

[25] Klinakis A, Lobry C, Abdel-Wahab O, Oh P, Haeno H, et al (2011) A novel tumour-suppressor function for the Notch pathway in myeloid leukaemia. Nature 473: 230–233. 21562564 10.1038/nature09999 PMC3093658

