# Supplementary material for: Epigenetic Inactivation of Notch-Hes Pathway in Human B-Cell Acute Lymphoblastic Leukemia
Source: PLoS One. 2013 Apr 26;8(4):e61807. doi: 10.1371/journal.pone.0061807 (PMC3637323; doi:10.1371/journal.pone.0061807)
Supplement: Figure S1 — Expression of Notch3, JAG1, Hes4 and Hes2 in normal bone marrow (BM), CD34+ BM, BMs from patients with T cell acute lymphoblastic leukemia (T-ALL), B-ALL, and various leukemia cell lines. The relative gene expression was determined by real-time PCR assays and normalized to that of GAPDH. (PPT) [file pone.0061807.s001.ppt]

## Slide 1
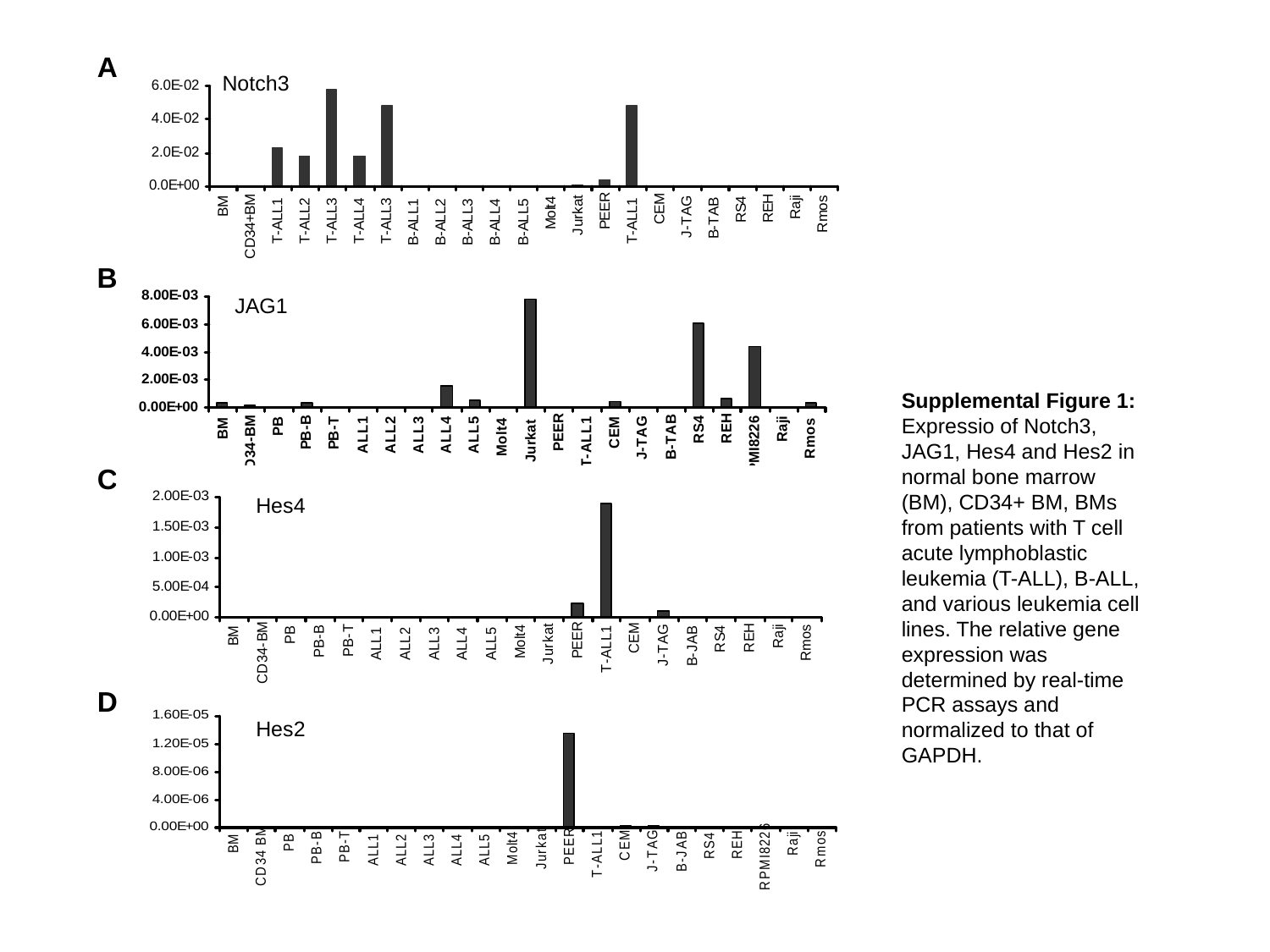

A
Notch3
B
JAG1
Supplemental Figure 1: Expressio of Notch3, JAG1, Hes4 and Hes2 in normal bone marrow (BM), CD34+ BM, BMs from patients with T cell acute lymphoblastic leukemia (T-ALL), B-ALL, and various leukemia cell lines. The relative gene expression was determined by real-time PCR assays and normalized to that of GAPDH.
C
Hes4
D
Hes2
