# Supplementary material for: Epigenetic Inactivation of Notch-Hes Pathway in Human B-Cell Acute Lymphoblastic Leukemia
Source: PLoS One. 2013 Apr 26;8(4):e61807. doi: 10.1371/journal.pone.0061807 (PMC3637323; doi:10.1371/journal.pone.0061807)
Supplement: Figure S2 — Notch3, Hes4, Hes2 and Hes6 expression levels in various leukemia cell lines. The leukemia cells were either untreated, or treated with 5-aza-2′-deoxycytidine (DAC) only, suberoylanilide hydroxamic acid (SAHA) only or both (D+S) as described in material and methods. Real-time PCR analysis. In general, expression of Notch3 and Hes4 was restored in some leukemia cell lines treated by DAC with or without SAHA. Hes2 was un-respond to any DAC, and SAHA treatment. In contrast, Hes6 was respond to DAC, and SAHA treatment. (PPT) [file pone.0061807.s002.ppt]

## Slide 1
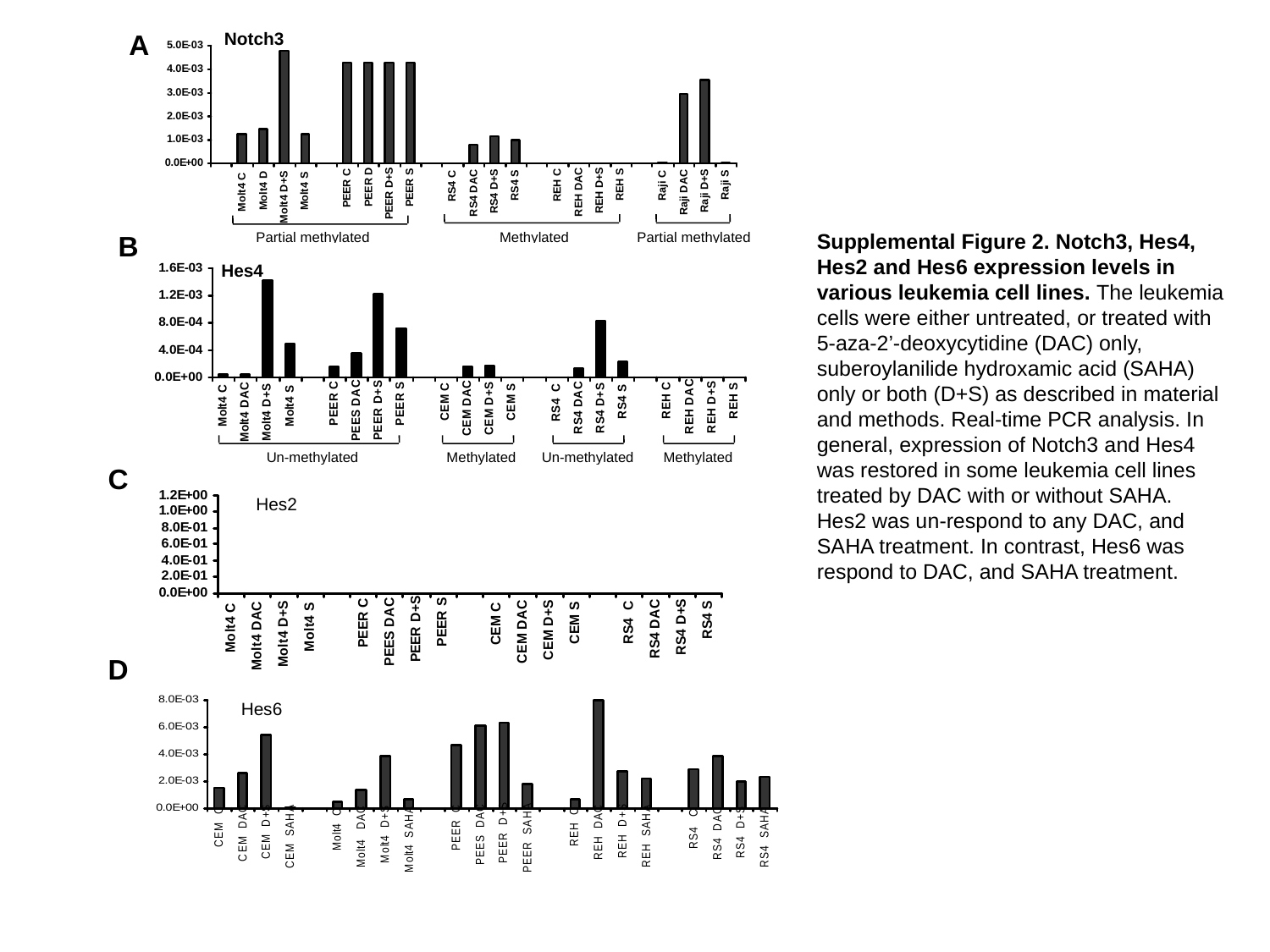

A
Notch3
Partial methylated
Methylated
Partial methylated
B
Hes4
Un-methylated
Methylated
Un-methylated
Methylated
C
Hes2
D
Hes6
Supplemental Figure 2. Notch3, Hes4, Hes2 and Hes6 expression levels in various leukemia cell lines. The leukemia cells were either untreated, or treated with 5-aza-2’-deoxycytidine (DAC) only, suberoylanilide hydroxamic acid (SAHA) only or both (D+S) as described in material and methods. Real-time PCR analysis. In general, expression of Notch3 and Hes4 was restored in some leukemia cell lines treated by DAC with or without SAHA. Hes2 was un-respond to any DAC, and SAHA treatment. In contrast, Hes6 was respond to DAC, and SAHA treatment.
