# Supplementary material for: Epigenetic Inactivation of Notch-Hes Pathway in Human B-Cell Acute Lymphoblastic Leukemia
Source: PLoS One. 2013 Apr 26;8(4):e61807. doi: 10.1371/journal.pone.0061807 (PMC3637323; doi:10.1371/journal.pone.0061807)
Supplement: Figure S3 — A. FUGW lentiviral constructs for transducing Hes5 and controls. B. Western blot analysis. Hes5 expression was detected in untreated T-ALL1 cells, as well as 293T and TALL1 cells transduced with Hes5. (PPT) [file pone.0061807.s003.ppt]

## Slide 1
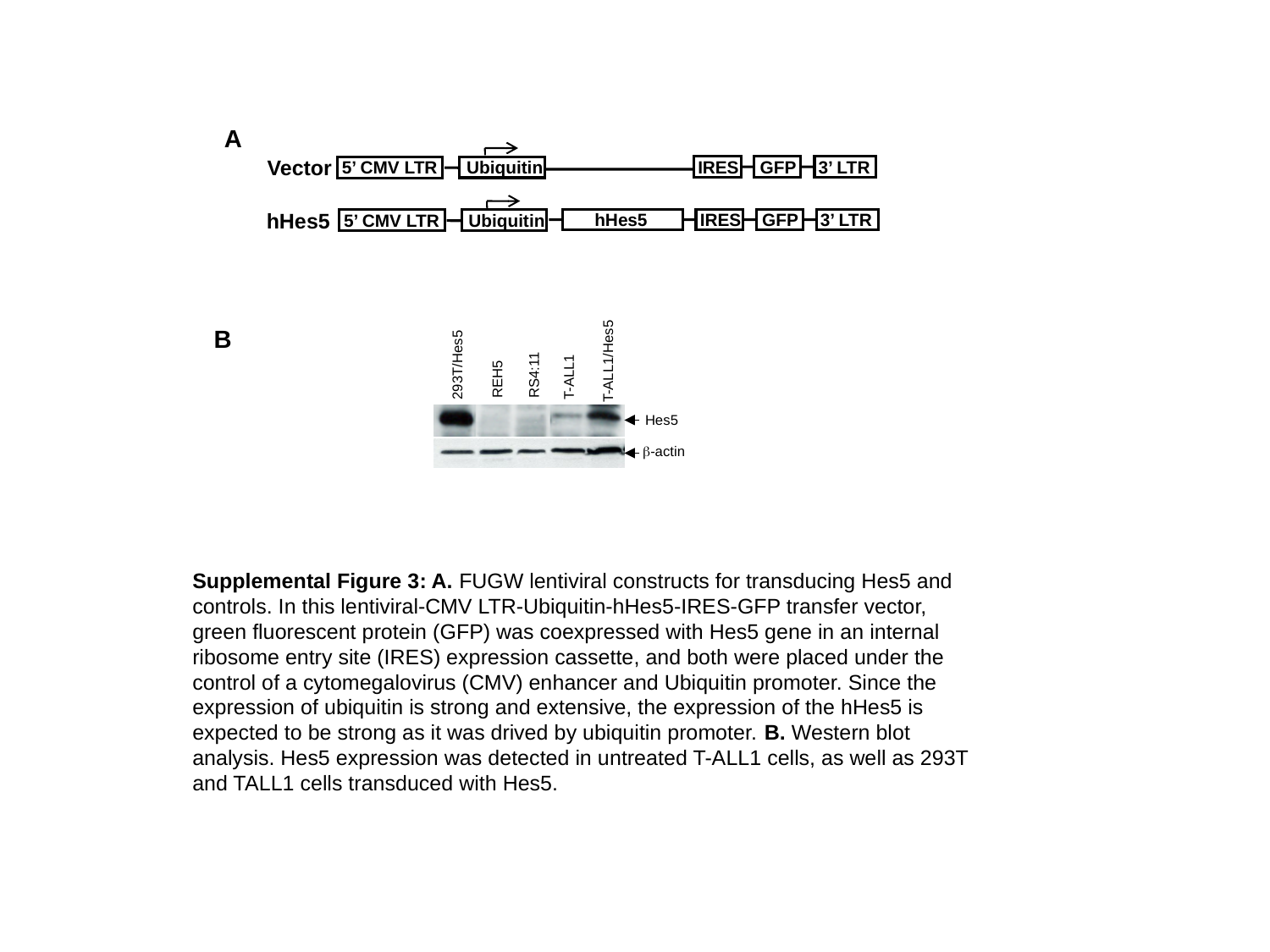

A
Vector
IRES
GFP
3’ LTR
5’ CMV LTR
Ubiquitin
hHes5
 hHes5
IRES
GFP
3’ LTR
5’ CMV LTR
Ubiquitin
T-ALL1/Hes5
293T/Hes5
RS4:11
T-ALL1
REH5
Hes5
β-actin
B
Supplemental Figure 3: A. FUGW lentiviral constructs for transducing Hes5 and controls. In this lentiviral-CMV LTR-Ubiquitin-hHes5-IRES-GFP transfer vector, green fluorescent protein (GFP) was coexpressed with Hes5 gene in an internal ribosome entry site (IRES) expression cassette, and both were placed under the control of a cytomegalovirus (CMV) enhancer and Ubiquitin promoter. Since the expression of ubiquitin is strong and extensive, the expression of the hHes5 is expected to be strong as it was drived by ubiquitin promoter. B. Western blot analysis. Hes5 expression was detected in untreated T-ALL1 cells, as well as 293T and TALL1 cells transduced with Hes5.
