# Supplementary figures and images for: Epigenetic Inactivation of Notch-Hes Pathway in Human B-Cell Acute Lymphoblastic Leukemia
Source: PLoS One. 2013 Apr 26;8(4):e61807. doi: 10.1371/journal.pone.0061807 (PMC3637323; doi:10.1371/journal.pone.0061807)

## Slide 1
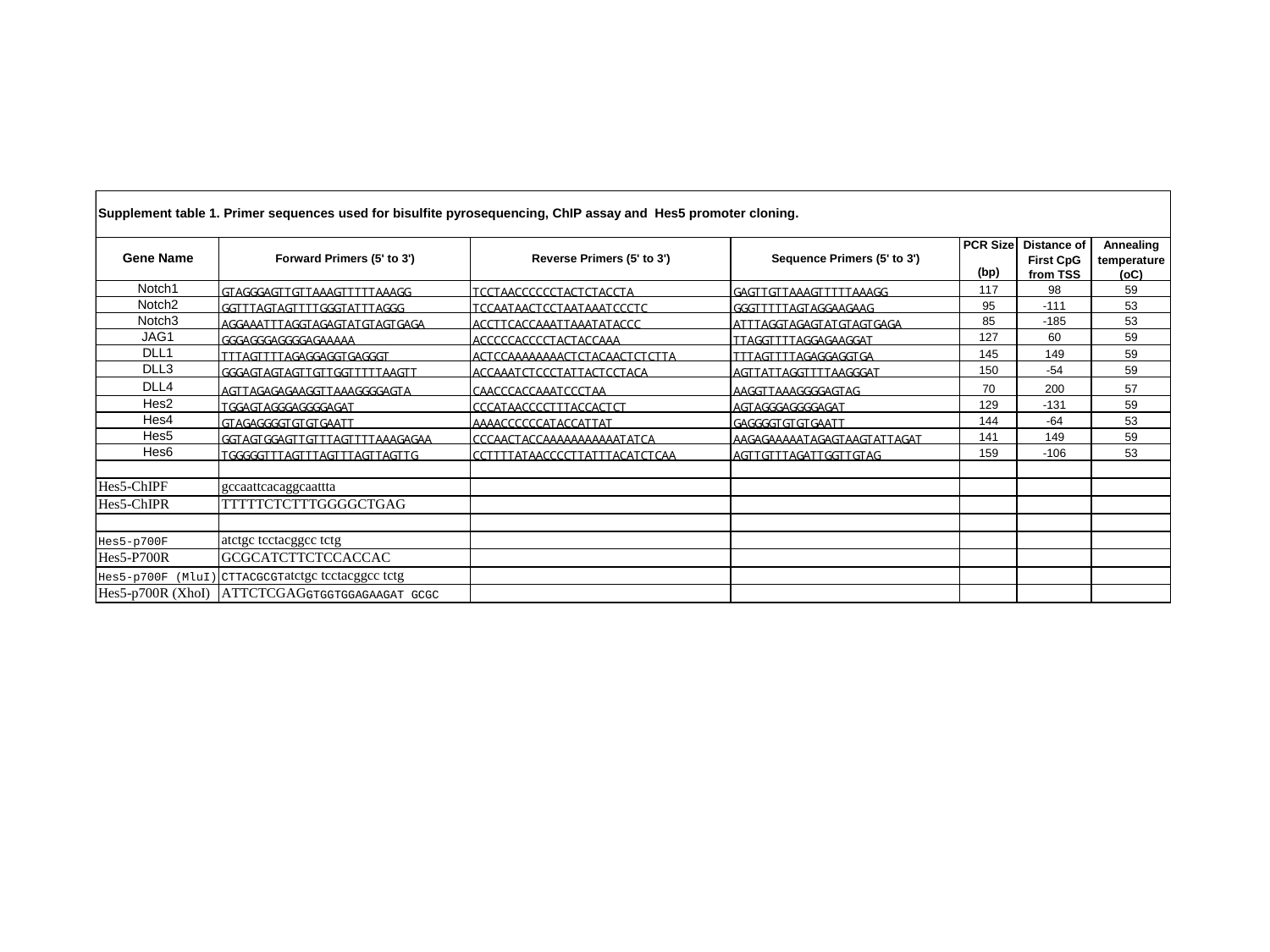

Supplement: Table S1 — Primer sequences used for bisulfite pyrosequencing, ChIP assay and Hes5 promoter cloning. (PPT) [file pone.0061807.s004.ppt]
